# Suprapubic Osteomyelitis in an Intravenous Drug User: A Case Report

**DOI:** 10.7759/cureus.21312

**Published:** 2022-01-17

**Authors:** Karimah Best, Siham Hussien, Atika Malik, Salauni Patel, Miriam B Michael

**Affiliations:** 1 Internal Medicine, American University of Antigua, Osbourn, ATG; 2 Internal Medicine, University of Maryland Midtown Campus, Baltimore, USA; 3 Internal Medicine, Punjab Hospital, Sialkot, PAK; 4 Internal Medicine, Howard University, Washington DC, USA; 5 Internal Medicine, University of Maryland, Baltimore, USA

**Keywords:** methicillin-resistant staphylococcus aureus (mrsa), cocaine, pubic symphysis, intravenous drug use (ivdu), pubic osteomyelitis

## Abstract

We discuss a case of methicillin-resistant *Staphylococcus* *aureus* (MRSA) osteomyelitis pubis in a 45-year-old female patient with an active history of intravenous (IV) drug injection. While IV drug users are typically infected with *Pseudomonas aeruginosa* in cases of osteomyelitis of the pubic symphysis, our patient presented with a rare case of MRSA infection of the pubis symphysis. In this case, an investigation using magnetic resonance imaging (MRI), elevated levels of erythrocyte sedimentation rate (ESR), C-reactive protein (CRP), and culture was consistent with the diagnosis of osteomyelitis. Osteomyelitis pubis is an infection that causes necrosis and destruction of the pubic bone. This condition remains a rarity, as less than 1% of osteomyelitis cases are reported to involve the pubic symphysis, thus contributing to the delays observed between onset of symptoms and diagnosis. The goal of this case report is to promote awareness of this phenomenon to hasten diagnosis and early treatment. The recommended treatment is with IV antibiotics for MRSA coverage for four to six weeks’ duration; however, our patient left against medical advice.

## Introduction

Osteomyelitis of the pubis symphysis is an infectious process of the bone that is seen as inflammatory changes within the bone on magnetic resonance imaging (MRI) and/or computed tomography (CT), and erythrocyte sedimentation rate (ESR) and C-reactive protein (CRP) are often elevated [1]. It often presents as a severe subacute pubic pain that is exacerbated by weight bearing and active movements such as walking. Such localized musculoskeletal pain in addition to an elevated ESR makes this diagnosis likely [1,2].

We describe the case of a 45-year-old female with an active history of IV drug use with injection into her mons pubis region, leading to the development of pubic symphysis osteomyelitis. The patient has undergone incision and drainage, started on intravenous (IV) antibiotics to cover for methicillin-resistant *Staphylococcus aureus* (MRSA), and failed to complete a full course of treatment as she left against medical advice.

While IV drug users are typically infected with *Pseudomonas aeruginosa* in cases of osteomyelitis of the pubic symphysis [1], athletes are mostly infected with Staphylococcus aureus. However, our patient is a rare case of IV drug user infected with MRSA in the pubis symphysis region.

Patients who have a history of IV abuse are susceptible to antibiotic-resistant infections, such as MRSA, and a resultant complicated treatment course. Providers should have a high suspicion of osteomyelitis of the pubic symphysis if patients present with pubic pain, difficulty in ambulating, and an elevated ESR.

## Case presentation

We present a case of a 45-year-old female with a medical history significant for polysubstance abuse consisting of opioids, cocaine, alcohol, and benzodiazepines, a hepatitis C infection, and an active chronic history of IV use who presented to the Emergency Department (ED) complaining of pain, swelling, and drainage in her mons pubis. The symptoms had been occurring for a two-week period prior to admission, with the pain progressively worsening causing her to have difficulty with ambulation, hence prompting her to seek medical assistance. The patient endorsed ongoing severe polysubstance abuse and injecting cocaine and heroin into her groin. The patient denied fever, chills, shortness of breath, nausea, vomiting, dysuria, diarrhea, or vaginal discharge. She denied recent trauma or sexual activity. Physical examination was notable for hemodynamic stability as the patient was afebrile with a temperature 97.7 degrees Fahrenheit, normotensive with a blood pressure of 118/82 mmHg, normal heart rate of 90 beats per minute, and eupneic with a respiratory rate of 15 breaths per minute and an oxygen of 97% on room air. Her pudendal area appeared swollen, with mild tenderness on palpation. There was no identifiable fluid collection, no erythema, and no bloody discharge. Laboratory results were remarkable for a white blood cell count of 11.6 K/mcL (normal range: 4.0-11.0 K/mcL), ESR of 95 mm/hour (normal range for females: 0-20 mm/hour), CRP of 1.80 mg/dL (normal range: 0.8-1.0 mg/dL), blood urea nitrogen of 40 mg/dL (normal range: 7-18 mg/dL), creatinine of 5.09 mg/dL (normal range: 0.6-1.2 mg/dL), and hypokalemia 2.8 mmol/L (normal range: 3.5-5.0 mol/L). Urinary toxicology screen was positive for cocaine, fentanyl, and oxycodone/oxymorphone. Of note, the patient was prescribed 10 mg oxycodone every four to six hours, as needed, for pain.

In the ED, the patient was started on vancomycin and piperacillin-tazobactam. The patient was stabilized and admitted to the medicine floor for further diagnostic imaging and management.

**Figure 1:**
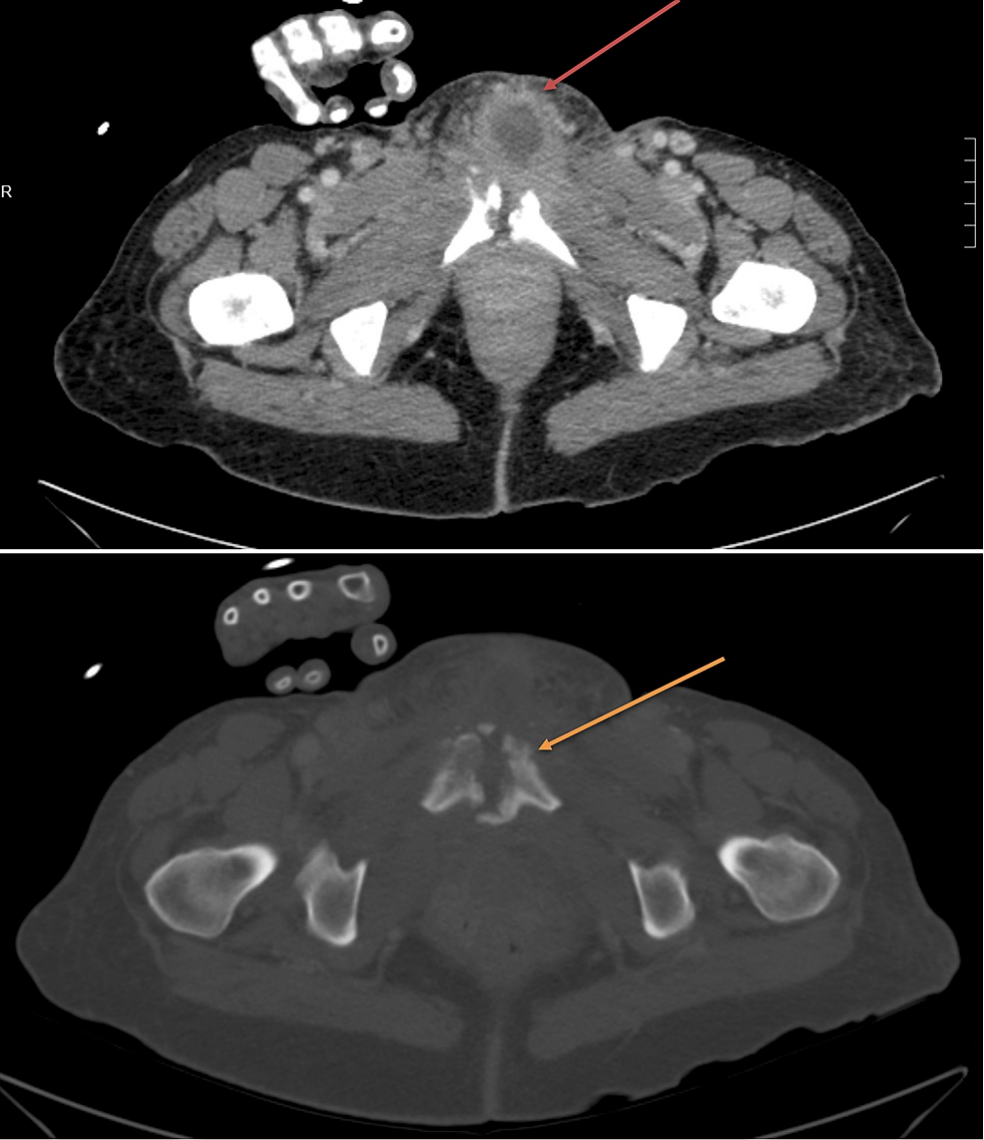
Venous-phase CT of the abdomen and pelvis. Red arrow indicates large overlying phlegmonous changes and anterior multi-loculated abscess, and orange arrow indicates destruction of the pubic symphysis with cortical erosions. Soft tissue swelling anterior and posterior to the pubic symphysis consistent with septic arthritis was also noted.

On the medicine floor, to assess the progression of the pelvic infection, computed tomography (CT) without contrast was planned due to the patient's setting of acute kidney injury. CT showed persistent fluid collection of the mons pubis with possible draining sinus tract extending into the pubic symphysis. Increasing osseous destruction of the pubic bone was noted along with reactive dystrophic soft tissue calcifications (Figure 1). The evidence was concerning an active infection with chronic osteomyelitis and septic arthritis. Initially, general surgery was consulted and recommended management via pack wounds with iodoform gauze, continuation of the broad-spectrum antibiotics, and consultation with orthopedic surgery and infectious disease.

**Figure 2:**
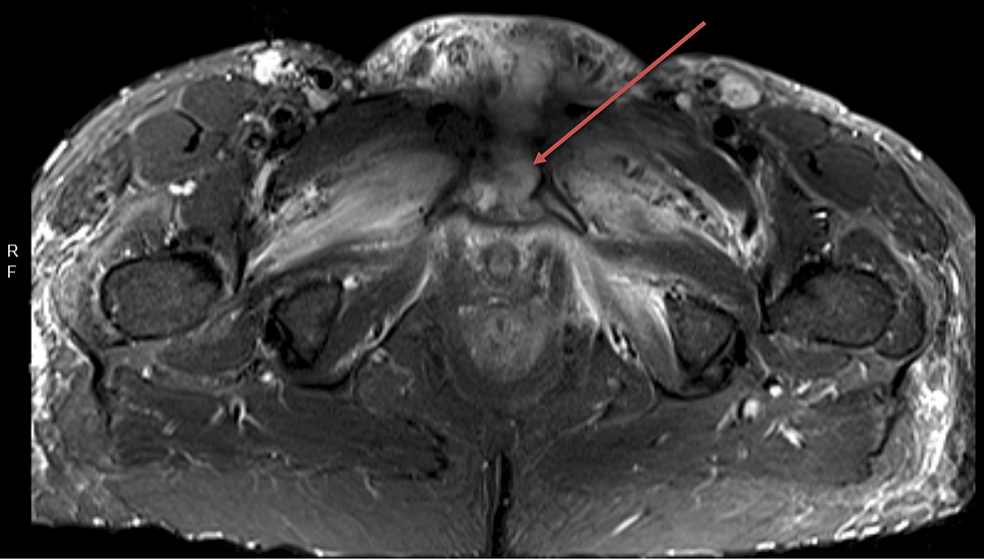
Short-T1 inversion recovery (STIR) MRI of the pelvis. Red arrow indicates destructive changes of the symphysis pubis with osteomyelitis of the pubic bodies.

The patient underwent surgical incision and drainage, intraoperative wound cultures returned positive for MRSA, and the patient was transitioned to a renal dosage of piperacillin-tazobactam 2.25 g every six hours, vancomycin was discontinued, and linezolid 600 mg IV every 12 hours was started. Prior to discharge, a peripherally inserted central catheter line was placed for continuation of IV antibiotics for another four to six weeks. However, the patient was lost to follow-up. Nineteen days later, the patient returned to the ED complaining of pelvic pain. Given the patient's history, diagnostic imaging of MRI was conducted, which revealed pubic symphysis osteomyelitis with an abscess straddling with destroyed joint spaces. Myositis and fasciitis of the surrounding structures was evident. (Figure 2). The patient was once again lost to follow-up as she left against medical advice.

## Discussion

We present a case of MRSA osteomyelitis of the pubic symphysis in a patient with IV injection of heroin and cocaine into the pubic region. While the pathogenesis remains unclear [1], we speculate that the infectious process occurred from hematogenous spread or direct inoculation from the needle. IV drug use can predispose a patient to infectious complications presumably from a combination of altered immune competence and injection of contaminated material [2].

This condition remains a rarity, as less than 1% of osteomyelitis cases are reported to involve the pubic symphysis [3], thus contributing to the delays observed between symptom onset and diagnosis [4].

The clinical presentation of an infectious cause compared to an inflammatory cause of the suprapubic pain overlap. It is important to distinguish between the two, as the course of treatment is different. Providers often confuse osteitis pubis, a self-limited inflammatory condition, with osteomyelitis pubis, an infectious process that can cause necrosis and destruction of the bone. Osteitis is often secondary to overuse injuries often seen in athletes. Osteomyelitis is often due to postoperative inoculation or after gynecological, urological operation, or endoscopic inguinal hernia repair [1].

There should be high clinical suspicion for osteomyelitis if a patient presents with severe subacute pubic pain that is exacerbated by weight bearing and active movements such as walking. This localized musculoskeletal pain in addition to an elevated ESR, makes this diagnosis likely [1,2]. Of the 13 reported cases of infectious pubis in IV drug user patients, temperature and leukocyte count were normal or slightly elevated [2]. In a review of 99 cases of septic arthritis of the pubic symphysis, WBC count was normal in 65% of patients and the ESR was high [4].

The pubic pain that patients experience often radiates to the groin, thigh, and/or hip because the hip adductors insert onto the pubic symphysis. Thus, involvement of the hip adductor accounts for pain experienced with ambulation [4], as exhibited in this patient. IV drug users of a younger age are more predisposed to septic arthritis of symphysis pubis due to the laxity of the ligament, as it is bound by flexible fibrocartilage with thin layers of hyaline cartilage [1,4]. In comparison, older patients have sclerosed and ossified joints, which reduce the risk of bacteremia [4].

While IV drug users are typically infected with *Pseudomonas aeruginosa* in cases of osteomyelitis of the pubic symphysis, [4], athletes are mostly infected with Staphylococcus aureus. However, our patient is a rare case of IV drug user infected with MRSA in the pubis symphysis region.

While our patient was lost to follow-up, in case reports reviewed by Magarian and Reuler, it has been shown that patients who followed up after discharge and completed a prolonged four-to six-week course of antibiotics did not exhibit recurrence or require debridement [2]. Often, chronic cases of osteomyelitis necessitate surgical debridement [5,6], as increasing prevalence of antibiotic-resistance organisms such as MRSA complicates antimicrobial therapy options [6].

To ensure targeted treatment, direct sampling of the bone for culture and sensitivity is essential. Our patient’s culture returned MRSA samples, and to treat MRSA osteomyelitis, vancomycin 1 g IV every 12 hours is the first-line therapy option [5]. However, since vancomycin has been associated with nephrotoxicity in some patients, linezolid 600 mg IV every 12 hours is indicated in patients presenting with abnormal kidney function [5,7,8].

## Conclusions

Providers should have a clinical suspicion of osteomyelitis of the pubic symphysis if a patient presents with pubic pain, difficulty in ambulating, and an elevated ESR on laboratory studies. Scientific investigations report that this condition often occurs postoperatively; however, it is of upmost importance to recognize that this infectious process can occur in patients who partake in IV drug use. This patient population is susceptible to infection with *Pseudomonas aeruginosa*; however, our patient highlights the possibility of MRSA infection. Suprapubic osteomyelitis in IV drug users is a rarity; thus, awareness of this phenomenon can aid in diagnosis and early targeted treatment to prevent a complicated treatment course.
